# Yttrium-90 radioembolization of isolated hepatic adrenocortical carcinoma metastases with negative surgical pathology

**DOI:** 10.1186/s13550-021-00755-0

**Published:** 2021-02-18

**Authors:** Sen Lu, Jasreman Dhillon, Julie Hallanger Johnson, Ghassan El-Haddad

**Affiliations:** 1 Department of Diagnostic and Interventional Radiology, University of South Florida, 2 Tampa General Circle, STC 7028, Tampa, FL 33606 USA; 2 Department of Pathology, Moffitt Cancer Center, 12902 USF Magnolia Drive, Tampa, FL 33612 USA; 3 Department of Endocrinology, Moffitt Cancer Center, 12902 USF Magnolia Drive, Tampa, FL 33612 USA; 4 Department of Diagnostic Imaging and Interventional Radiology, Moffitt Cancer Center, 12902 USF Magnolia Drive, Tampa, FL 33612 USA

**Keywords:** Adrenocortical carcinoma, Yttrium-90, Radioembolization, Flow redistribution, Surgical pathology, Adrenal crisis

## Abstract

**Background:**

Adrenocortical carcinoma (ACC) is an uncommon malignancy with an estimated 15,400 new cases annually across the globe. The prognosis is generally poor as the disease is often already advanced at initial diagnosis due to non-specific symptoms. Even for local disease, recurrence after surgical resection is high. Treatment choices for advanced disease include mitotane, chemotherapy, ablation, chemoembolization, radioembolization, and external beam radiotherapy, with varying degrees of efficacy. To the best of our knowledge, there have only been two prior case studies of complete clinical and radiological response of stage 4 disease at 1 year and 2 years after yttrium-90 (^90^Y) microsphere selective internal radiation therapy (SIRT) of isolated hepatic metastases post-surgery and chemotherapy.

**Case presentation:**

We present a case of a 58-year-old man with metastatic ACC who was treated with ^90^Y resin microsphere (SIR-spheres) for local control of liver metastases leading to a surgically proven negative pathology after partial hepatectomy 7 months after SIRT. The patient was initially diagnosed with stage 1 ACC that progressed 6 years later to stage 4 disease with new liver metastases that were deemed unresectable at an outside institution. After review of the case at multidisciplinary tumor board, he was referred for liver directed therapy for local tumor control. Angiographic workup demonstrated partial extrahepatic supply to the tumors from the right inferior phrenic artery, which was successfully embolized on the day of SIRT for flow redistribution. As the patient was being treated with mitotane that suppresses steroid production, he developed post-SIRT adrenal crisis, which was successfully controlled with steroids, highlighting the need for pre SIRT stress dose steroids.

**Conclusions:**

This case continues to add to the literature supporting ^90^Y radioembolization as an effective treatment for isolated hepatic ACC metastases. Our case is the first to demonstrate surgically proven negative pathology after radioembolization. Further prospective study is warranted to better establish efficacy as well as safety of SIRT for ACC liver metastases.

## Background

Adrenocortical carcinoma (ACC) affects an estimated 0.5 – 2 new cases per million people annually, with a median survival of less than one year for stage 4 disease [1–3]. Overall, the median 5-year survival is 16–44% [4]. The prognosis is considerably better for stage 1 with a 5-year survival of 60%, compared to 0% for stage 4 [1, 2]. However, 70% of patients are initially diagnosed with stage 3 or 4 due to non-specific presentations, most commonly of excess hormone secretion (especially hypercortisolism or Cushing syndrome) or symptoms secondary to mass effect (e.g., abdominal or back pain, nausea, or vomiting) [1, 3]. Treatment of local disease involves surgical resection, but this is infrequently curative, with an 80% recurrence rate even after complete resection [1, 2]. Radiofrequency ablation has demonstrated some success for primary unresectable ACC < 5 cm [1, 5, 6]. Transcatheter arterial chemoembolization (TACE) has been reported with favorable outcomes for isolated hepatic metastatic lesions < 3 cm [1, 7]. To date, there has only been two case reports demonstrating efficacy of ^90^Y radioembolization for hepatic ACC metastases by follow-up imaging [2, 4]. Three additional cases of ^90^Y SIRT from a single institution demonstrated prolonged overall survival in patients with hepatic ACC metastases when analyzed in combination with 2 cases of liver-directed therapy treated with TACE [8]. We present a case of successful treatment of isolated hepatic adrenocortical carcinoma metastases, with the benefit of definitive negative surgical pathology after ^90^Y radioembolization.

## Case presentation

**Fig. 1:**
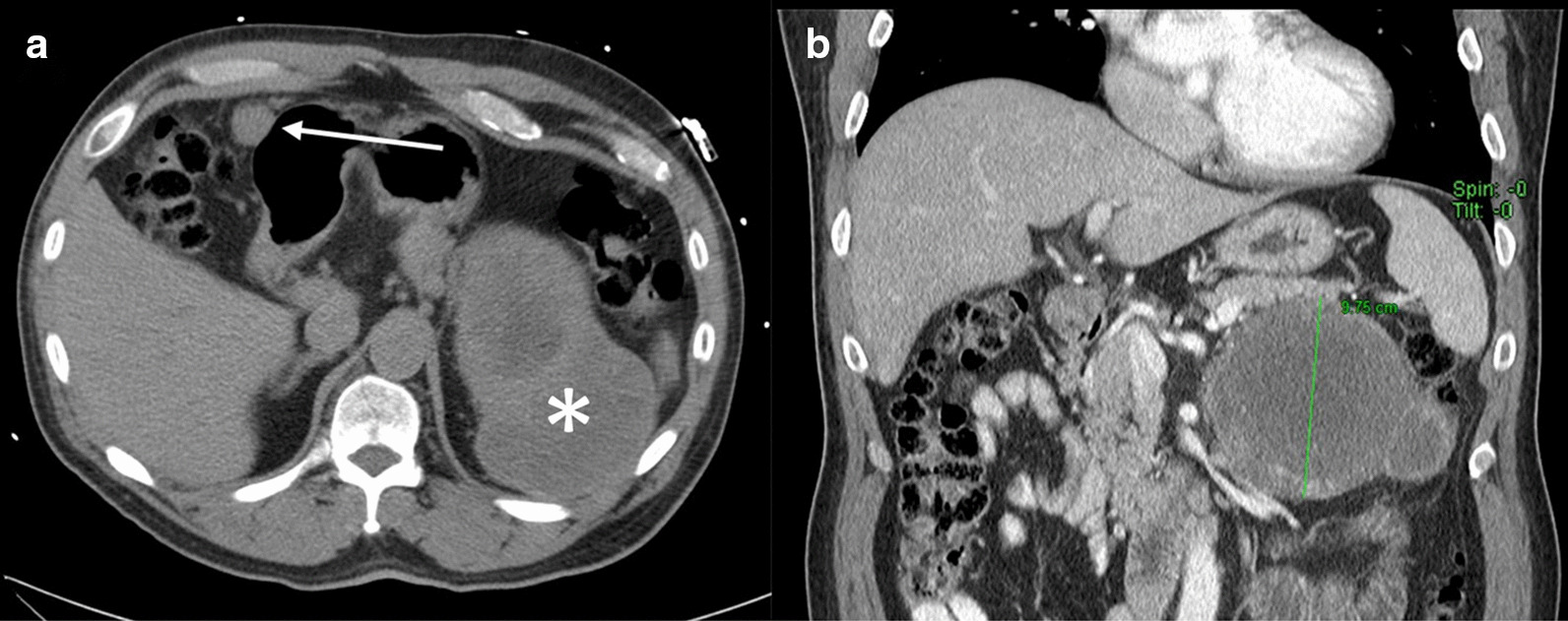
Incidental left adrenal mass. **a** Non-contrast CT chest demonstrated a large, heterogeneous left suprarenal mass (asterisk), and a 3 cm gastric nodule (arrow). **b** Contrast-enhanced CT demonstrated a predominantly necrotic 13.8 × 9.8 × 9.4 cm left suprarenal mass with scattered areas of enhancement and central low-attenuation, with subsequent pathology demonstrating adrenocortical carcinoma

A 58-year-old male with no significant past medical history presented to an outside institution with vague fatigue and left upper quadrant pain. A CT scan revealed a 15 ×9 × 9 cm left retroperitoneal mass as well as a 3 cm gastric tumor (Fig. 1). Biopsy of the retroperitoneal mass was consistent with adrenocortical carcinoma. He underwent a left adrenalectomy and partial gastrectomy with pathology confirming ACC and a gastrointestinal stromal tumor (GIST) of the stomach. He then began mitotane, an adrenolytic, for 2.5 years and adjuvant radiation to the adrenal bed.

**Fig. 2:**
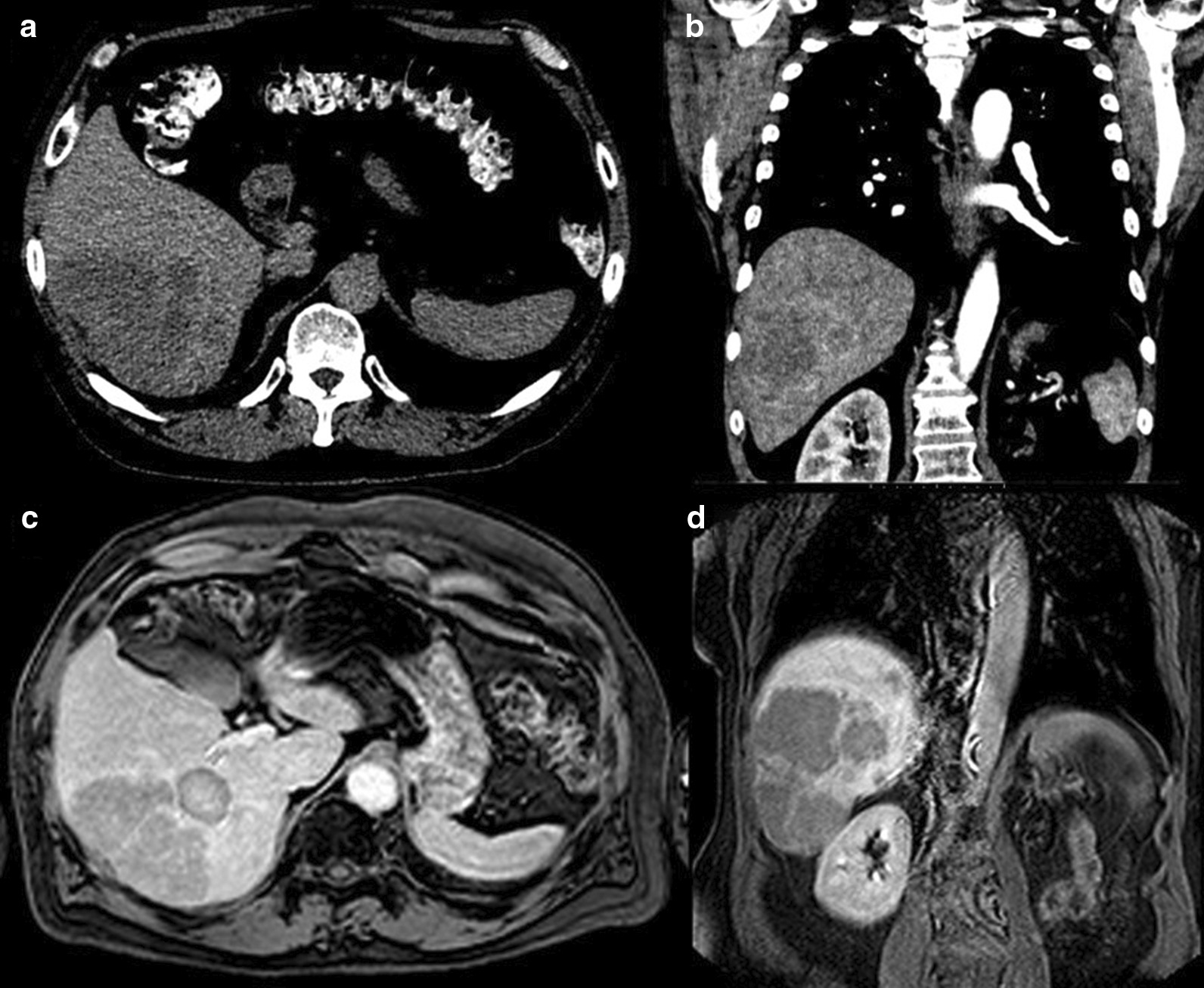
Metastatic adrenocortical carcinoma. **a** Six years after initial diagnosis, a surveillance non-contrast CT showed a lobulated, hypodense 9.5 × 8.7 cm hepatic segment 5/6 mass. **b** Contrast-enhanced CT showed peripheral enhancement with central necrosis, suspicious for metastases. **c** 3-min post-Eovist (Bayer, New Jersey, USA) T1-weighted fat-suppressed MRI showed multiple ring-enhancing right inferior hepatic lesions. **d** One month after attempted surgical resection, delayed post-Eovist T1-weighted fat-suppressed MRI showed enlarging right inferior hepatic masses

His surveillance was performed at outside institutions, and his scans remained negative for the following 4 years. A follow-up surveillance CT scan done 6 years after diagnosis revealed a 9.5 × 8.7 cm right hepatic mass. This was followed with an MRI at that time that confirmed the presence of suspicious liver metastases (Fig. 2). Right hepatectomy was attempted at an outside institution but aborted due to the suspected presence of “miliary disease” based on the surgeon’s visualization of small plaque-like lesions on the liver surface. Therefore, only a segment 5 hepatic nodule was resected for permanent section and genetic sequencing and a cholecystectomy was performed. Review of the patient’s available images from that time showed no left hepatic lobe lesions visible on imaging. Per the outside surgery report, the patient did receive an [^18^F]Fluorodeoxyglucose ([^18^F]FDG) positron emission tomography/computed tomography (PET/CT) in addition to the MRI prior to surgery, which showed no extrahepatic disease (images not available).

**Fig. 3:**
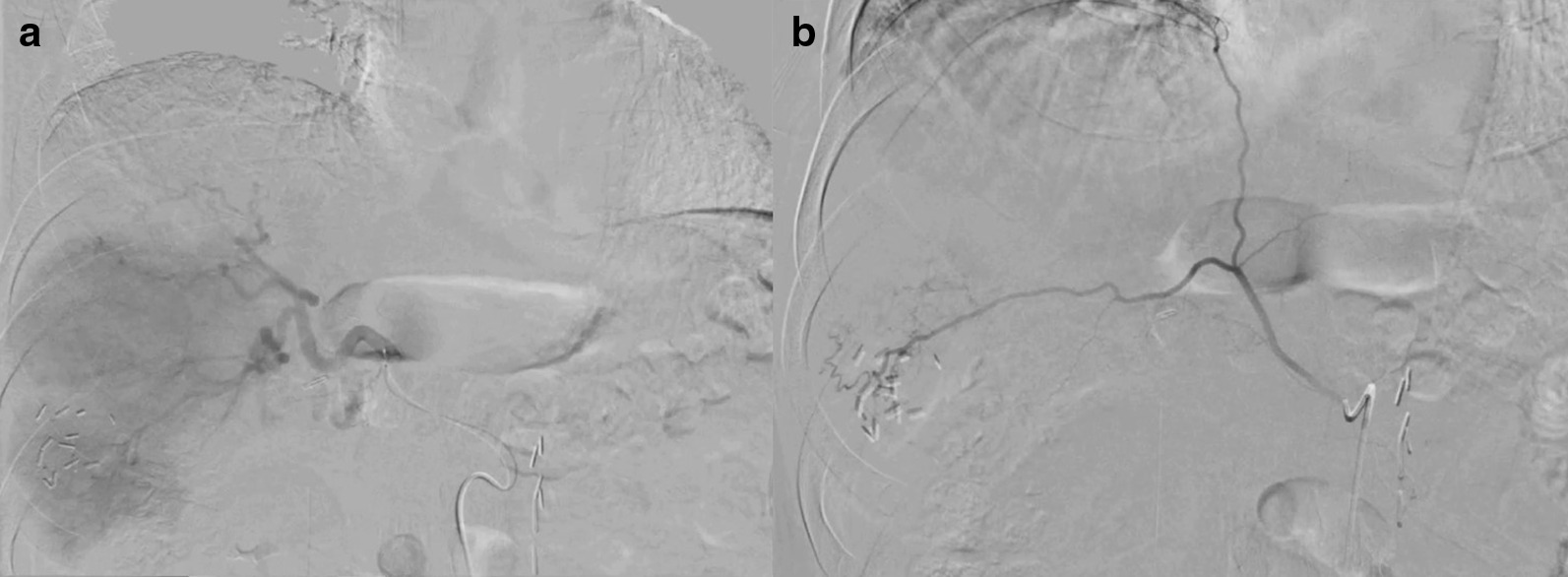
Angiographic workup for radioembolization. Hepatic arteriography demonstrated multiple hypervascular tumors supplied predominantly from right hepatic artery branches (**a**) and to a lesser extent by a branch of the right inferior phrenic artery (**b**). Post-surgical clips from prior attempted resection are seen in the inferior right hepatic lobe, which correlated to segment 5 on cross-sectional imaging

**Fig. 4:**
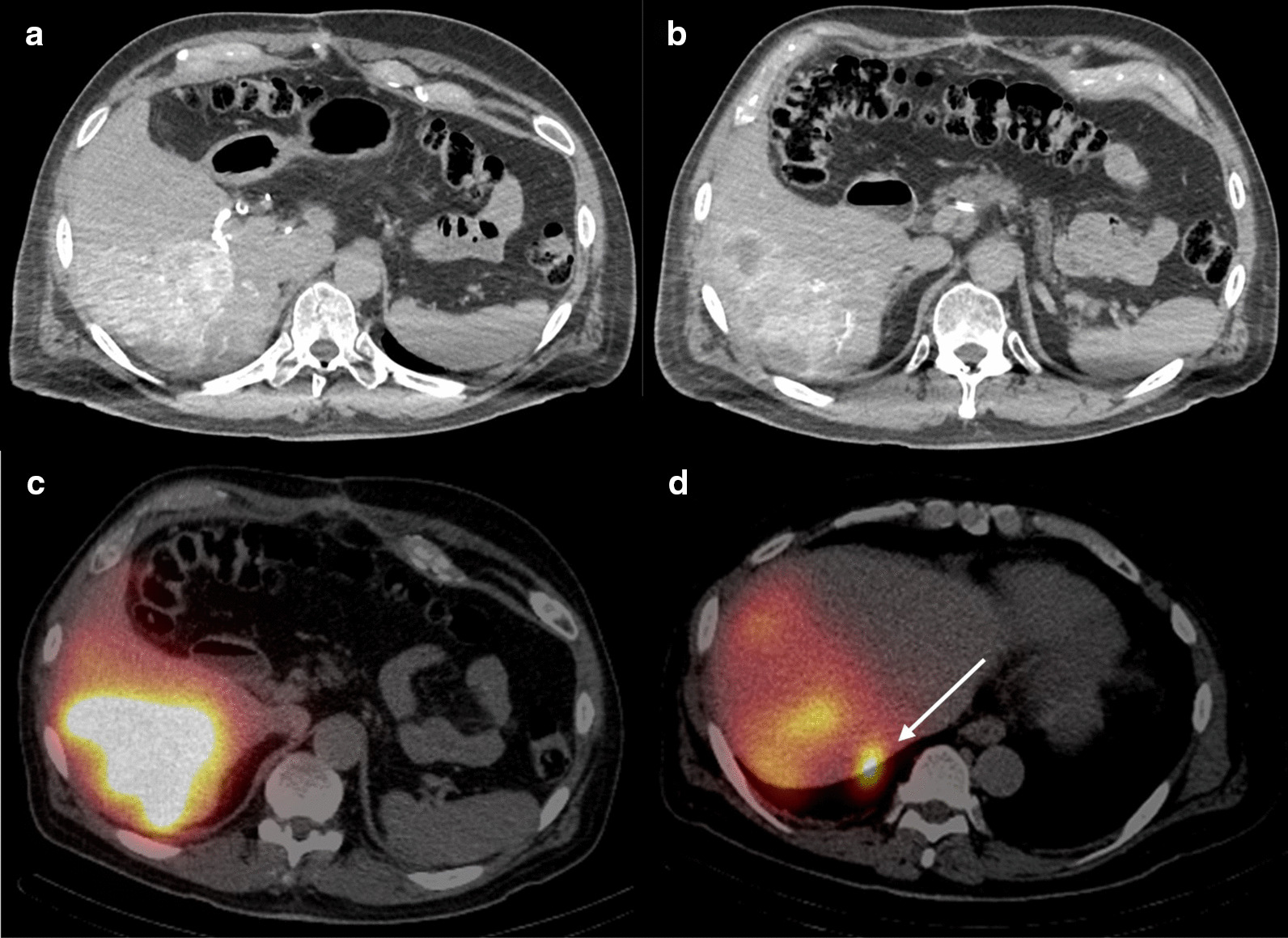
Axial images from pre ^90^Y 3D CT angiogram. **a** Intraprocedural selective arterial-phase contrast-enhanced CT of the right hepatic artery obtained during workup arteriography demonstrates hyperenhancing tumors with **b** hypoenhancing lesions along the posterior inferior liver, thought to be metastases supplied by branches of the right inferior phrenic artery. **c** SPECT/CT images after administration of [^99m^Tc]Tc-MAA into the right hepatic artery demonstrates intense activity within the majority of tumors in the inferior right hepatic lobe with photopenic peripheral areas that correlate to the hypoenhancing areas seen on 3D CT angiogram. **d** SPECT/CT images also demonstrate an area of intense focal activity along the posterior surface of hepatic segment VII (arrow), representing subserosal liver metastasis which may correspond to the plaque-like lesions seen during aborted initial surgery

After discussion of the case at our multidisciplinary tumor board, the decision was made for systemic therapy as well as liver directed therapy. The patient started an EDP chemotherapy schedule (etoposide, doxorubicin, and cisplatin). He then underwent visceral angiogram workup which demonstrated collateral supply to a portion of the right hepatic lobe tumors by right inferior phrenic artery branch (Fig. 3). A hepatopulmonary shunt of 3% was observed on planar scintigraphy and single-photon emission computed tomography (SPECT) (Fig. 4) after administration of 214.6 MBq [^99m^Tc]technetium-macroaggregated albumin ([^99m^Tc]Tc-MAA, Drax Image MAA kit; 99.7% purity) into the right hepatic artery. The hepatic volumes were manually calculated using the native basic segmentation tool from General Electric’s Picture Archiving and Communication Systems: total 2084 cm^3^, volume of liver to be treated 1537 cm^3^, and volume of tumor in the region 654 cm^3^. As there is paucity of data on what is the most appropriate dose to deliver to these tumors, a modified BSA model calculated the required activity at 1.8 GBq using an estimated lung mass of 1 kg. However, since these tumors can be radioresistant, we decided to increase the activity to 2 GBq. This would have given an estimated dose of 105 Gy to the tumor by partition model using a T/N ratio of 4, 26 Gy to the normal liver, and 3 Gy to the lungs.

**Fig. 5:**
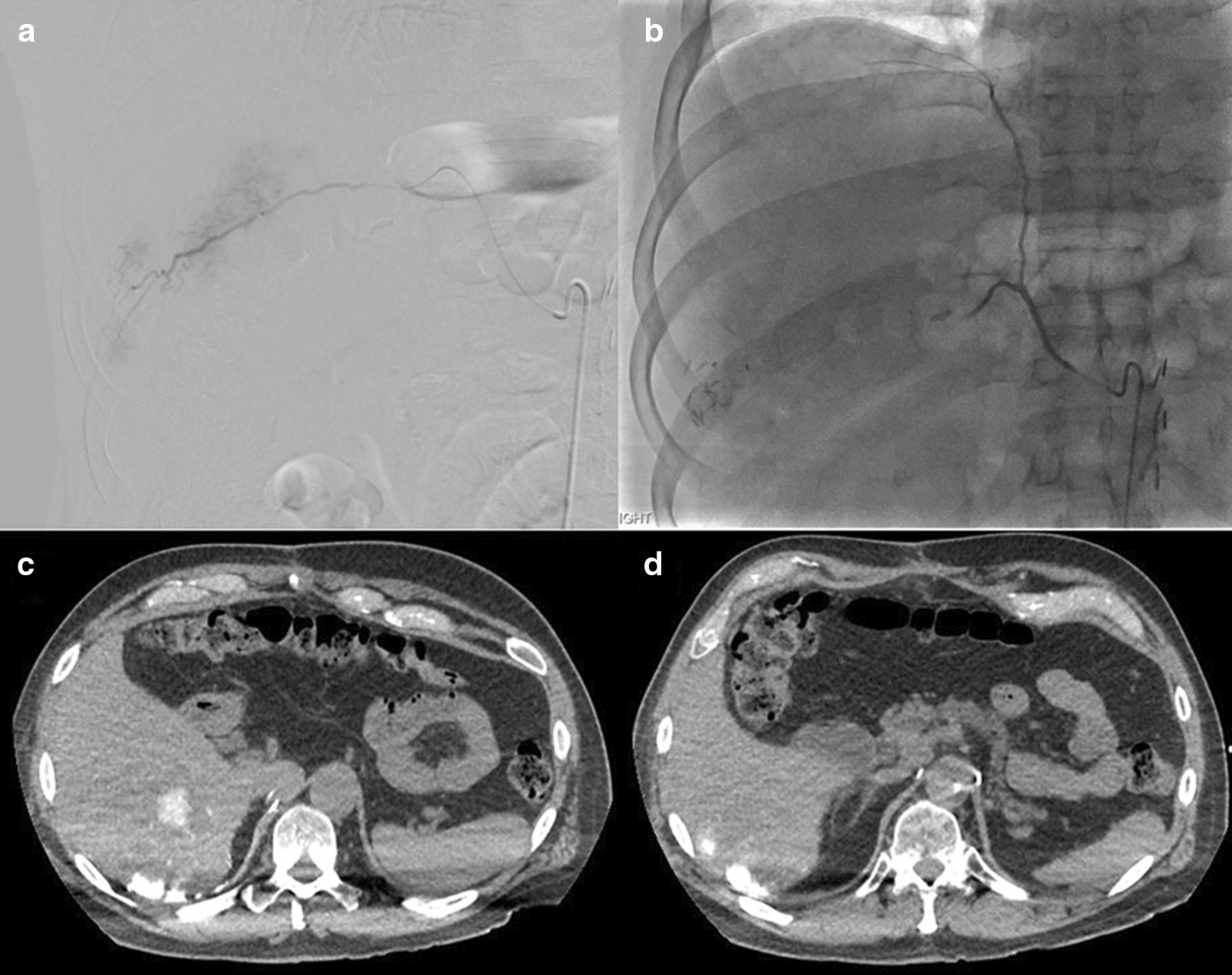
Right inferior phrenic artery. **a** The inferior branch of the right inferior phrenic artery was superselected, demonstrating tumor blush in the inferior right hepatic lobe. **b** Post-bland embolization of the inferior branch of the right inferior phrenic artery demonstrated stasis with persistent flow to the remaining branches of the right inferior phrenic artery. **c**, **d** Intraprocedural selective 3D contrast-enhanced CT angiogram of the inferior branch of the right inferior phrenic artery demonstrated enhancement of hepatic lesions along the posterior periphery of the right hepatic lobe

**Fig. 6:**
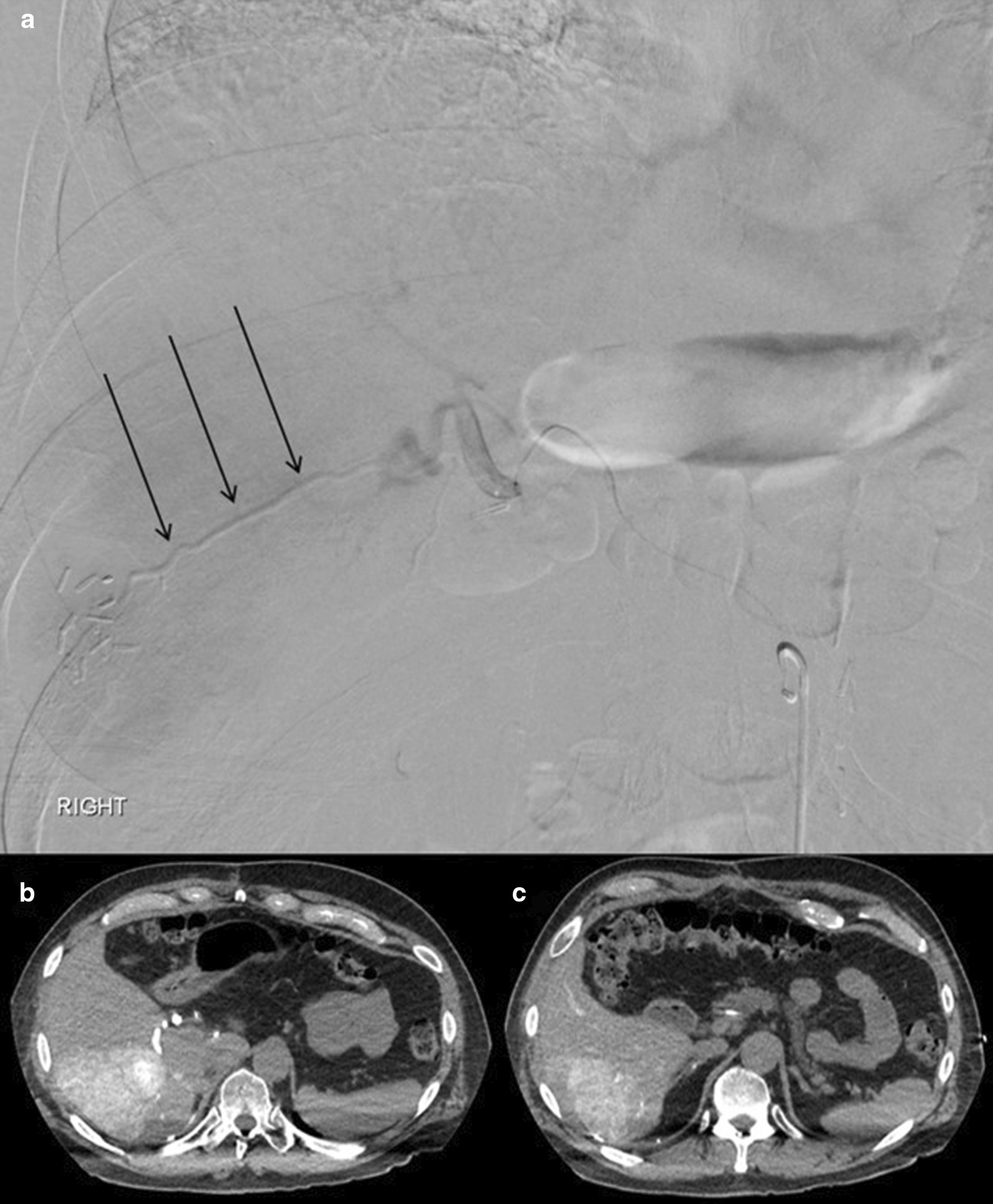
Pre-SIRT redistribution of flow. **a** Right hepatic artery arteriogram demonstrates the embolized inferior branch of the right phrenic artery, compatible with embolic material (arrows). **b**, **c** Intraprocedural selective 3D arterial-phase contrast-enhanced CT of the right hepatic artery demonstrates enhancement of the right hepatic lesions with perfusion of the tumors from the right hepatic artery instead of the right inferior phrenic artery branch that was bland embolized

On the procedure day, the patient first underwent bland embolization of the inferior branch of the right inferior phrenic artery using 900 µm Embosphere microspheres (Merit, Utah, USA) to cause redistribution of flow into the tumors along the surface of the right hepatic lobe from the right hepatic artery (Fig. 5). Subsequent ^90^Y SIR-sphere (Sirtex Medical Inc., Massachusetts, USA) resin embolization of the right hepatic artery was performed (Fig. 6) with a total administered activity to the perfused volume of 2.11 GBq. The patient developed post-procedure adrenal crisis in the recovery room manifested by abdominal pain and diaphoresis, and was treated with 20 mg of dexamethasone followed by 60 mg intravenous hydrocortisone every 6 h. After admission and steroid therapy, he was then discharged in stable condition on post-procedure day 3.

**Fig. 7:**
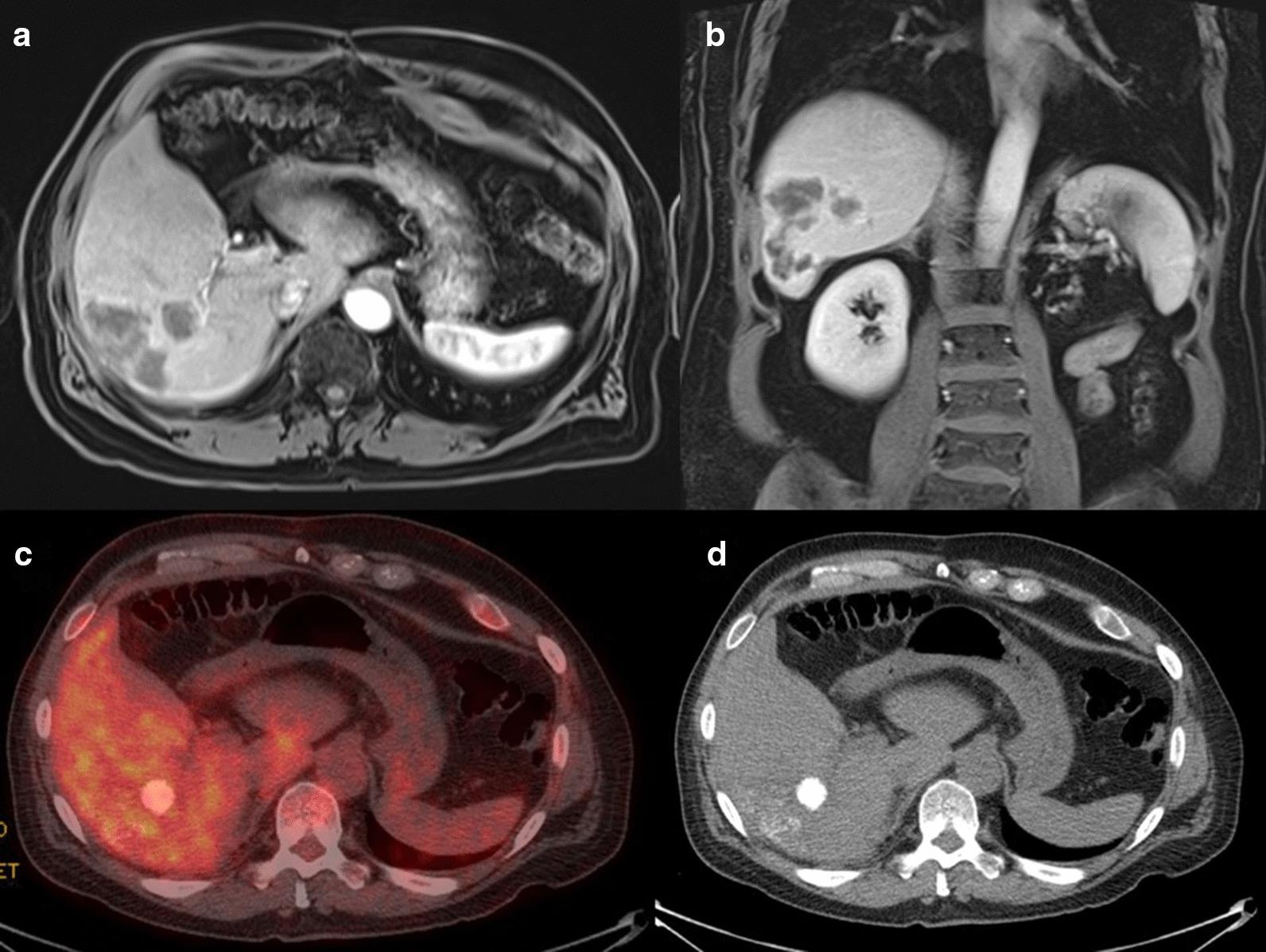
Post-radioembolization response. **a**, **b** 1-month post-radioembolization arterial phase post-Eovist T1-weighted fat-suppressed MRI shows decreased size of right hepatic lesions with no abnormal enhancement. **c**, **d** 3-month post-radioembolization PET/CT with [^18^F]FDG showed no residual activity in the right inferior hepatic tumor bed

**Fig. 8:**
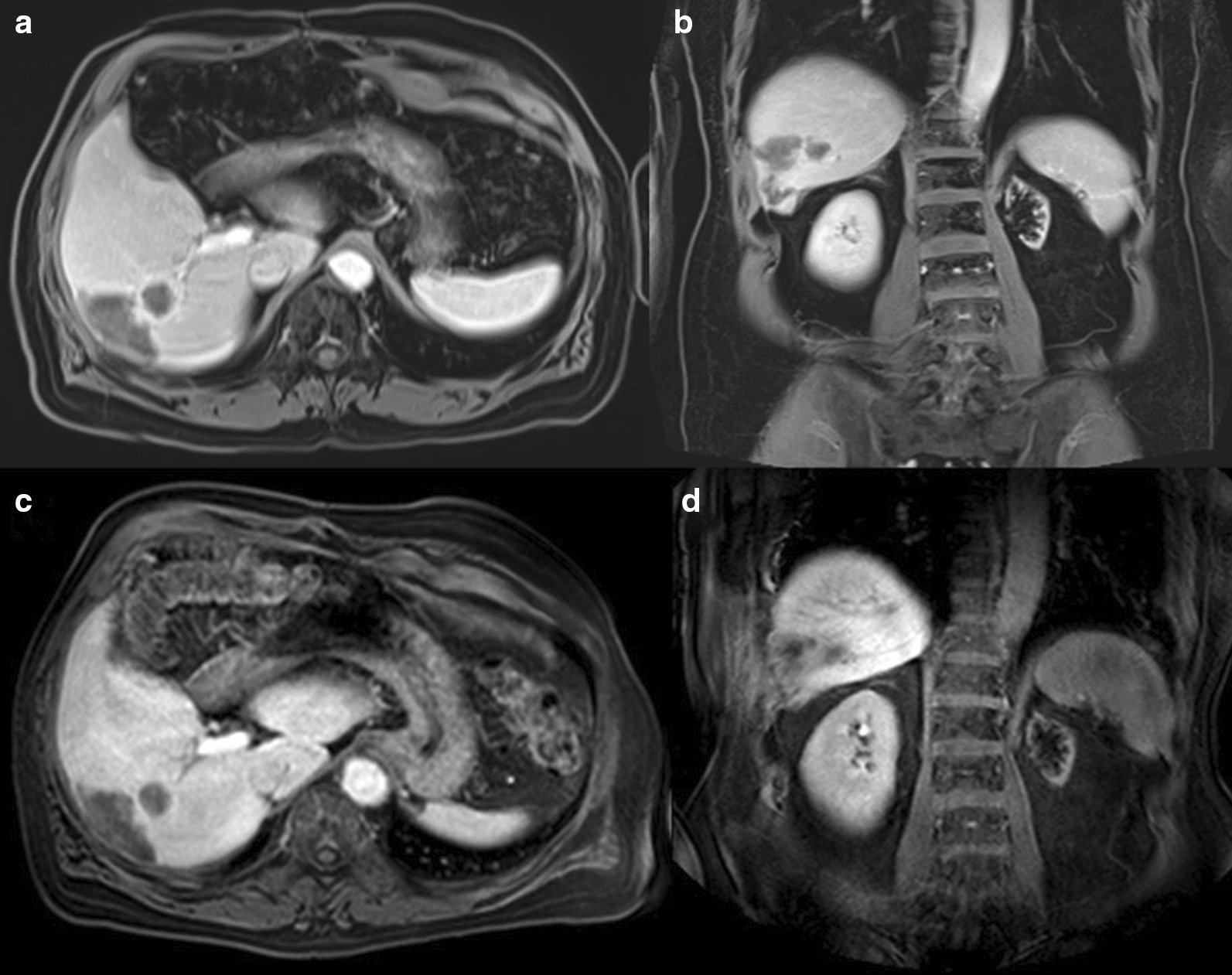
Continued post-radioembolization response. **a**, **b** Portal venous phase post-Eovist T1-weighted fat-suppressed MRI taken at 4 months and **c**, **d** 5 months post-radioembolization shows continued decrease in size of inferior right hepatic lesions

**Fig. 9:**
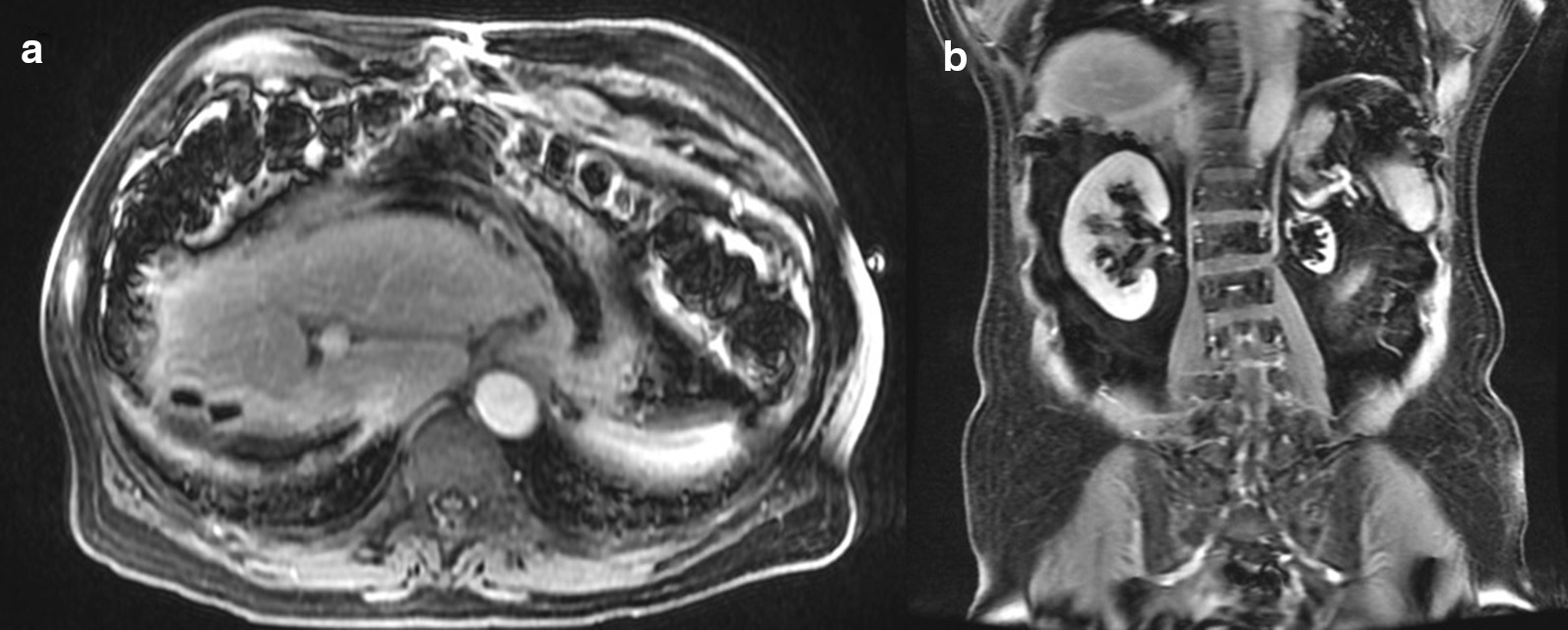
No recurrent disease on MRI 1-month post-partial right hepatectomy. **a** Axial and **b** coronal portal venous phase post-Eovist T1-weighted MRI images demonstrate blooming artifact from surgical clips in the right hepatic resection margin. Pathology did not demonstrate any viable tumor

The patient’s 1-month follow-up MRI showed decrease in the size of the multiple right hepatic metastatic lesions including the small lesions on the liver surface with no new sites of disease. Specifically, no lesions were seen in the left hepatic lobe. 3-month post-radioembolization [^18^F]FDG PET/CT showed no residual or recurrent  disease (Fig. 7). Four- and 5-month post-radioembolization MRI showed continued decrease in size and enhancement of the right hepatic lesions (Fig. 8). Despite the reassuring imaging findings, the patient requested surgical resection of the right hepatic lesions as the data is sparse on the effect of SIRT on ACC hepatic metastases. In conjunction with the multidisciplinary tumor board, surgery was deemed reasonable as the disease was limited to the right hepatic lobe and no miliary disease was seen on imaging. Hence, 7 months after radioembolization, he had partial right hepatectomy at an outside institution with pathology demonstrating no viable tumor including in the resection margin. One-month post-resection MRI showed no residual hepatic lesions (Fig. 9). The patient was in observation and surveillance with regular lab work for adrenal insufficiency, including renin, dehydroepiandrosterone sulfate, and adrenocorticotropic hormone. He declined adjuvant mitotane due to quality-of-life concerns and previous side effects. An incidental periportal lymph node measuring 1-cm short axis was found on CT chest 8 months post-resection (15 months post-radioembolization), which was subsequently found to be metastatic ACC on surgical pathology. Although he developed metastatic periportal lymphadenopathy, the patient is currently 16 months post-surgical resection and 23 months post-radioembolization with no hepatic recurrence.

## Conclusions

The goal of local treatment for ACC is curative, but this is only achieved 20% of the time. Local control with radiofrequency ablation for unresectable tumors or poor surgical candidates has been demonstrated, although progression of metastatic disease is high with 11 out of 13 patients demonstrating recurrence in one study [5]. Options for systemic treatment of advanced ACC have been with an EDP-M schedule (etoposide, doxorubicin, and cisplatin plus mitotane) or a M-S schedule (mitotane plus streptozosin) [9]. These regimens have comparable overall survival (14.8 vs 12 months), but EDP-M has been favored due to improved response rates and progression-free survival (5 vs 2.1 months) [2, 10]. Non-invasive radiation therapy has demonstrated no survival benefit and no reduction in the risk of metastasis [11]. Locoregional radiation therapy and surgical debulking or resection of recurrence has traditionally been palliative in nature. TACE has been reported with favorable outcomes for isolated hepatic metastatic lesions < 3 cm or > 50% lipiodol uptake, with progression in only 5 out of 29 patients (17%) [1, 7].

To the best of our knowledge, ours is the 6^th^ known case for hepatic ACC metastases successfully treated with ^90^Y radioembolization in combination with chemotherapy, and the 3rd case report of complete clinical and radiological response of isolated hepatic metastases after chemotherapy and surgical resection/debulking of the primary lesion. Ours is, however, the 1st case that demonstrates post-radioembolization pathologic correlation showing no residual viable carcinoma upon resection at 7 months post-radioembolization. Our case also demonstrated that bland embolization of the right inferior phrenic artery that was feeding a portion of the metastatic tumor was an effective strategy for flow redistribution just prior to radioembolization of the right hepatic artery. The metastatic disease burden in our case was limited to the right hepatic lobe, and only one radioembolization procedure was necessary. Two prior cases demonstrated 12-month and 24-month radiologic and clinical response, respectively, and had bilobar disease thus requiring two separate ^90^Y radioembolizations [2, 4].

Compared to TACE, which requires patients to be admitted, ^90^Y radioembolization can be performed on an outpatient basis and is more cost-effective. Our case also provides a reminder that close attention should be paid to the clinical status of the patient in the intraprocedural or immediate post-procedure period for signs of adrenal crisis (hypotension, nausea, vomiting, abdominal pain, weakness, fatigue, lethargy, fever, confusion, or coma). Management of adrenal crisis includes volume repletion with normal saline (with or without 5% dextrose) and pre-emptive perioperative IV hydrocortisone. Due to the acuity and severity of the condition, treatment should not be delayed by diagnostic tests.

This case continues to add to the literature supporting ^90^Y radioembolization as an effective treatment for isolated hepatic ACC metastases. Our case demonstrates complete surgical pathology-proven response 7 months post-radioembolization. In addition, we utilized bland embolization to successfully redistribute flow from a feeding right inferior phrenic artery to the right hepatic artery for effective radioembolization. Our patient unfortunately developed metastatic lymphadenopathy in periportal lymph nodes first detected at 15 months post-radioembolization, but remains disease free in the liver. Further prospective study is warranted to better establish efficacy, as well as safety of SIRT for ACC liver metastases.

## Data Availability

Data sharing is not applicable to this article as no datasets were generated or analyzed during the current study.

## Abbreviations

[^18^F]FDG: [^18^F]Fluoro-2-deoxy-D-glucose
[^99m^Tc]Tc-MAA: [^99M^Tc]technetium-macroaggregated albumin
^90^Y: Yttrium-90
ACC: Adrenocortical carcinoma
cm: Centimeter
EDP: Etoposide, doxorubicin, and cisplatin
GBq: Gigabecquerel
GIST: Gastrointestinal stromal tumor
MBq: Megabecquerel
mg: Milligram
PET/CT: Positron emission tomography/computed tomography
PLT: Platelet
SIRT: Selective internal radiation therapy
SPECT: Single-photon emission computed tomography
TACE: Transcatheter arterial chemoembolization
